# Correspondence

**Published:** 1887-06

**Authors:** 


### CORRESPONDENCE.

CHICAGO, ILL., MAY 6TH, 1887.

Dr. Geo. H. Cushing,

Rec. Secy. American Dental Association,

Dear Sir:

Your letter of April 30th, accompanied with a copy of a communication from Drs. W. C. Barrett and Frank Abbott making a protest against a proposed action of the officers of the Association, looking to the postponement of the regular annual meeting of the American Dental Association of the present year, until 1888, and raising what they term a "point of order," as to the power of the officers, under the constitution to do so; also your letter of more recent date, with a statement of the vote upon the question submitted to the officers of the Association, is received.

As the question raised had not occurred to me before, and the existing circumstances are of more than ordinary importance, I have thought it best to take such time to consider the subject, as would enable me to reach a correct conclusion as to the power of the officers to change the time and place of the Association's annual meeting; hence the delay in my reply.

It should be noticed that the gentlemen do not question the power of the officers to change the time and place of the meeting, provided the time is fixed within the year 1887. The authority the officers have (if such authority exists) to postpone the next annual meeting to 1888 is found in Section two (2) of Article four (4) of the Constitution. So much of the article as in any way governs the question, and to which reference is made by the protestants reads as follows: "TIME OF MEETINGS.—The regular meeting of the Association shall be held annually, and commence on the first Tuesday in August. The place of meeting shall be determined each year by vote of the Association.

Sec. 2—The officers may for extraordinary reasons change the time and place of meeting, upon the written consent of ten (10) of the fifteen officers."

As Drs. Barrett and Abbott are not officers of the Association, and have no right to vote upon the question at issue, or upon an appeal from any decision made, I could not see clearly how they could raise a point of order. The question, it seemed to me (for the time being at least), was a matter that rested entirely with the officers of the Association. The point they raise is valuable however, as it has suggested a more thoughtful consideration of the question as to whether the officers have the power to *omit* a regular annual meeting of the Association, for if the regular meeting for this year is postponed until 1888, the meeting of 1887 or that of 1888 would of necessity have to be omitted.

Upon a careful reading of the article referred to, I became satisfied that an honest construction of its meaning forbade the officers postponing the meeting until next year. But before giving this as my decision, I felt that I should take counsel with some one in whose opinion the Association could rest with assured confidence. With this idea in view, I placed the Constitution of the Association, and the protests of Drs. Barrett and Abbott, in the hands of Ex-United States Senator Lyman Trumbull, and asked him to give me his written opinion upon the entire subject, which will be found in the following copy of a letter received from him:

CHICAGO, MAY 4TH, 1887.

Dr. W. W. Allport,

President American Dental Association,

Argyle Building, City:

Dear Sir—The authority of the officers of the American Dental Association to dispense with the annual meeting is one of power under the constitution, and not a question of order as to the course of proceeding, which could only be raised by a member of the Association in one of its meetings. In my opinion a fair construction of Article 4 of the Association's constitution, requires regular meetings of the Association to be held annually. While Section 2 of that article authorizes the officers, for extraordinary reasons, to change the time and place of meeting upon the written consent of ten of the fifteen officers, I do not think it contemplated a repeal of the first section, which requires annual meetings to be held, but was intended rather to authorize a change of the time and place of holding the annual meeting.

My conclusion is that the officers would not be authorized under the constitution to dispense altogether with the annual meeting.

Yours Truly,

LYMAN TRMMBULL.

With the opinion of a gentleman of such large experience in, and accurate knowledge of, parliamentary procedure, and acknowledged standing as an able constitutional lawyer, I must accept his interpretation, and decide that the officers of the Association have no power to *omit* an annual meeting, and therefore direct that the vote just taken be not recorded.

Very Truly Yours,

DR. W. W. ALLPORT, President.

